# Integration of Localized, Contextual, and Hierarchical Features in Deep Learning for Improved Skin Lesion Classification

**DOI:** 10.3390/diagnostics14131338

**Published:** 2024-06-24

**Authors:** Karthik Ramamurthy, Illakiya Thayumanaswamy, Menaka Radhakrishnan, Daehan Won, Sindhia Lingaswamy

**Affiliations:** 1 Centre for Cyber Physical Systems, Vellore Institute of Technology, Chennai 600127, India; menaka.r@vit.ac.in; 2 Department of Computational Intelligence, School of Computing, SRM Institute of Science and Technology, Kattankulathur 603203, India; illakiyt@srmist.edu.in; 3 System Sciences and Industrial Engineering, Binghamton University, Binghamton, NY 13902, USA; dhwon@binghamton.edu; 4 Department of Computer Applications, National Institute of Technology, Tiruchirappalli 620015, India; sindhia@nitt.edu

**Keywords:** dermoscopic images, deep learning, skin cancer, convolutional neural network, image processing

## Abstract

Skin lesion classification is vital for the early detection and diagnosis of skin diseases, facilitating timely intervention and treatment. However, existing classification methods face challenges in managing complex information and long-range dependencies in dermoscopic images. Therefore, this research aims to enhance the feature representation by incorporating local, global, and hierarchical features to improve the performance of skin lesion classification. We introduce a novel dual-track deep learning (DL) model in this research for skin lesion classification. The first track utilizes a modified Densenet-169 architecture that incorporates a Coordinate Attention Module (CoAM). The second track employs a customized convolutional neural network (CNN) comprising a Feature Pyramid Network (FPN) and Global Context Network (GCN) to capture multiscale features and global contextual information. The local features from the first track and the global features from second track are used for precise localization and modeling of the long-range dependencies. By leveraging these architectural advancements within the DenseNet framework, the proposed neural network achieved better performance compared to previous approaches. The network was trained and validated using the HAM10000 dataset, achieving a classification accuracy of 93.2%.

## 1. Introduction

Skin lesions are localized irregularities of the skin, characterized by atypical growths or discolorations. It can result from factors such as prolonged sun exposure, genetic susceptibility, infections, and environmental pollutants. The lesions include both benign (moles, warts) and malignant (melanoma) types [1]. Skin cancer is the most common form of cancer worldwide. Skin cancer poses a significant public health concern worldwide, with millions of non-melanoma and melanoma cases reported each year. The incidence of both non-melanoma and melanoma skin cancers has been increasing over the past decades. Currently, between 2 and 3 million non-melanoma skin cancers and 132,000 melanoma skin cancers occur globally each year [2]. There is a significant rise in melanoma incidence rates across various countries and global regions. This emphasizes the critical need for early and accurate diagnosis to facilitate timely intervention and treatment. Therefore, accurate differential diagnosis of malignant cutaneous melanoma and its benign simulators is crucial for providing patients with optimal and successful treatment.

The clinical diagnosis of skin lesions presents significant challenges. This is primarily due to the inherent heterogeneity in the skin lesions, the frequent overlap of clinical features between benign and malignant lesions, and the inherent subjectivity in interpretation by clinicians. These factors can contribute to inconsistencies and potentially lead to misdiagnoses. Dermoscopic images have emerged as vital tools in aiding diagnosis by providing magnified views of the skin, thus revealing the structure and pigmentation patterns of the lesion. Technological advancements have led to progress in automated detection methods for skin cancer and the development of computer-aided diagnosis systems. These computational methodologies serve to augment the capabilities of dermatologists by facilitating the identification and classification of lesions, improving diagnostic accuracy and efficiency.

The advent of machine learning (ML) has significantly improved the field of skin lesion detection. ML algorithms have demonstrated promising potential in classifying skin lesions based on dermoscopic images. These algorithms frequently necessitate the manual extraction of features and possess restricted learning capabilities, potentially compromising their performance and reliability. These systems rely on the availability of high-quality data for training, which may pose challenges in datasets with limited diversity or size. Moreover, ML algorithms may struggle to generalize across different patient populations or account for evolving disease patterns, leading to potential biases or inaccuracies in diagnosis. The emergence of DL methodologies has allowed for the automatic learning of hierarchical features from raw data, making them highly useful for skin lesion identification. DL is a powerful alternative for diagnosing skin cancer, overcoming the limitations of traditional ML-based methods. Moreover, they provide improved scalability and adaptability, allowing for optimal performance with various datasets and patient groups. Deep learning methods can help resolve interpretability issues by assisting clinicians in comprehending the diagnostic results. Using DL in an automated diagnostic system can enhance the detection of benign and malignant skin lesions, enabling timely intervention to mitigate the serious consequences.

Although traditional DL approaches have strengths, they still have limitations. These techniques are prone to overfitting, especially when dealing with imbalanced datasets, which might cause the model to prioritize the dominant class [3]. These approaches also find it challenging to accurately represent spatial features and global connections in the input images. This research presents a novel two-track DL method for skin lesion classification, using the HAM10000 dataset. It incorporates a modified DenseNet-169 network and a customized CNN model. This dual-track approach leverages the strengths of DL to enhance the accuracy and reliability of skin cancer detection. The proposed model integrates the local and global features, which helps in precise localization and modeling of long-range dependencies. These features are crucial for distinguishing between benign and malignant lesions, thereby reducing the risk of misdiagnosis.

## 2. Literature Survey

Skin cancer is one of the most prevalent forms of cancer worldwide, with timely detection being essential for successful treatment. Over the past few decades, significant research efforts have focused on developing reliable methods for classifying and diagnosing skin lesions. Traditionally, this field has relied on expertise from dermatologists. However, recent advancements in Artificial Intelligence (AI), particularly DL techniques, have gathered considerable interest due to their performance and accuracy in skin lesion classification tasks. This section aims to provide an overview of the advancements in this field, specifically focusing on both ML and DL approaches utilized for skin lesion classification. Calderón et al. utilized a bilinear CNN method specifically designed for skin cancer detection [1]. The method demonstrated better results in classifying skin lesions based on dermatoscopic images, showing the potential of CNNs in this domain. Mehmood et al. introduced a shallower and broader Xception network optimized for the efficient classification of skin lesions [4]. Singh et al. presented a deep learning framework for skin lesion diagnosis that incorporates uncertainty estimation and explainability [5]. Rahman et al. proposed an ensemble learning-based approach for multiclass skin lesion classification, demonstrating the effectiveness of combining multiple models for improved performance [6]. Mporas et al. investigated traditional machine learning for skin cancer classification [7]. Their approach involved image pre-processing steps such as segmentation and hair removal. Among the classification algorithms evaluated, AdaBoost and random forest classifiers demonstrated better performance. Hoang et al. utilized a wide-ShuffleNet, which is a variant of ShuffleNet. It incorporates group convolution and channel shuffle operations for lightweight deep learning architectures. It also uses skip connections and batch normalization layers to enhance training efficiency and classification accuracy [8]. Tanzila et al. utilized an automated skin lesion detection and recognition approach using a deep CNN, incorporating contrast enhancement, lesion boundary extraction, and in-depth feature extraction with transfer learning [9]. Chaturvedi et al. conducted a comparative analysis on the performance of five contemporary CNNs for skin lesion classification [10]. The research utilized five pre-trained CNNs and four ensemble models for multi-class skin cancer classification. Modifications were made on architectures like Xception, InceptionV3, InceptionResNetV2, ResNeXt101, and NASNetLarge.

Maqsood and Damaševičius developed a framework for the identification and categorization of skin lesions using a DL approach that selects features showcasing the importance of feature engineering in achieving better classification results [11]. Salma and Eltrass used an automated deep learning method for distinguishing between skin lesions [12]. Techniques include morphological filtering for hair removal, Grab-cut for lesion segmentation, and investigation of pretrained CNN architectures like VGG-16, ResNet50, InceptionV3, and MobileNet for classification. Chatterjee et al. focused on extracting characteristics from cross-correlation in spatial and frequency domains for skin lesion categorization [13]. Their method leveraged both spatial and frequency domain information to improve classification accuracy. Shetty et al. employed ML and CNNs for skin lesion classification and demonstrated the benefits of combining traditional ML algorithms with DL techniques [14]. Fraiwan and Faouri employed deep transfer learning to automatically detect and classify skin cancer [15]. Their approach leveraged pre-trained models to achieve better accuracy with minimal training data, showcasing the potential of transfer learning in skin lesion classification. Jain et al. [16] and Huang et al. [17] also employed transfer learning-based approaches, demonstrating the effectiveness of leveraging pre-trained models for skin lesion classification.

Tajerain et al. employed a system that leverages an EfficientNet-B1 architecture followed by global average pooling and a 7-node softmax layer for classification [18]. Ali et al. focused on pre-processing, developing an image pipeline that removes hair from images, augments the dataset, and resizes images based on the specific requirements of EfficientNet models (B0–B7) [19]. Srinivasu et al. introduced a model that utilizes MobileNet V2 for skin disease classification, followed by a Long Short-Term Memory (LSTM) network to enhance performance [20]. Salamaa et al. investigated the use of ResNet50 and VGG-16 models, evaluating the impact of various pre-processing techniques and the inclusion or exclusion of a Support Vector Machine (SVM) for classification [21]. Himel et al. applied a vision transformer architecture for skin cancer classification [22]. This architecture utilizes a self-attention mechanism to extract spatial features within the image, enabling it to learn pertinent features critical for accurate lesion classification. Mehr et al. introduced a deep learning-based model that leverages a pre-trained Inception-ResNet-v2 network for image classification [23]. The study additionally incorporated patient data such as age, gender, and anatomical site to potentially improve classification accuracy.

Recent advancements in deep learning techniques have significantly improved the accuracy and efficiency of skin lesion classification. Feature extraction, selection, and fusion techniques, along with transfer learning and pre-trained models, have contributed to the improvement in classification accuracy. However, there is still a need for further research to address the challenges associated with the detection and classification of skin lesions, particularly in the context of early and accurate diagnosis of skin cancer.

### 2.1. Research Gaps

The purpose of this research is to address the following research gaps in the field of skin cancer classification:

1. While existing studies commonly utilize pre-trained models for skin cancer detection, these models may not consistently differentiate between benign and malignant lesions accurately. Therefore, there is a need to fine-tune pre-trained models specifically for skin lesion classification to achieve more precise results.
2. Previous research on skin cancer detection has predominantly focused on local features and their aggregation, neglecting the connections between distant regions in the images. This limited focus often leads to inadequate performance. Thus, there is a need to develop more advanced techniques capable of effectively capturing both local and global information from skin lesion images.
3. Despite the high accuracy demonstrated by CNNs in various skin cancer detection and classification tasks, their ability to capture complex information and manage long-range dependencies is often limited. This limitation can be problematic when addressing subtle disease symptoms that require a comprehensive understanding of the broader context.

### 2.2. Research Contributions

The research contributions of the proposed work are outlined as follows:

1. In first track, a modified DenseNet architecture is employed to capture local features. Concurrently, the FPN and GCN utilized in second track extract global features from the input images. The integration of the local and global features obtained from these two distinct tracks increases the overall performance of the proposed network.
2. By incorporating a custom CNN with DenseNet-169, the model harnesses the comprehensive knowledge embedded in state-of-the-art networks while also integrating task-specific adaptations from the custom network. This integrated approach results in enhanced performance and accelerated convergence during the training phase.
3. The proposed model utilizes the FPN for the extraction of hierarchical features, capturing fine-grained patterns within the image across multiple scales. Additionally, it integrates the GCN block, which captures non-local correlations and models contextual relationships within the channel-enhanced feature map derived from the FPN.

## 3. Materials and Methods

This section presents an outline of the materials and methodologies employed in the study. It encompasses the dataset description, the environmental setup used for the experiments, the data augmentation techniques applied, and details of the proposed deep learning model architecture.

### 3.1. Dataset Description

The Human Against Machine with 10,000 training images (HAM10000) dataset is a well-recognized and extensive collection of dermoscopic images designed for identifying skin lesions [24]. The dataset comprises a total of 10,015 dermoscopic images, which are categorized into seven distinct classes. These seven classes can be further categorized into malignant (cancerous) and benign (non-cancerous) groups. Specifically, the benign group includes 6705 images categorized as melanocytic nevi (nv), 1099 images as benign keratosis-like lesions (bkl), 142 images as vascular lesions (vasc), and 115 images classified under dermatofibroma (df). On the other hand, the malignant group consists of 514 images in the basal cell carcinoma (bcc) class, 327 images in the actinic keratoses (akiec) class, and 1113 images in the melanoma (mel) class.

### 3.2. Environment Setup

All experiments pertaining to the proposed network were conducted using computational resources provided by the Kaggle Cloud platform. The computational setup included a 16 GB Nvidia Tesla T4 GPU paired with a 30 GB CPU. The model was implemented using PyTorch, and the ADAM optimization algorithm was employed for model implementation. To address the issue of class imbalance and enhance the model’s discriminative capability, the focal loss function was utilized. An iterative process was employed to fine-tune the learning rate, number of epochs, batch size, dropout rate, and weight decay. Table 1 provides a comprehensive overview of the range of values explored during this optimization process. This optimization procedure significantly influences the performance of the model.

### 3.3. Data Augmentation

This section delineates the augmentation techniques employed to prepare the images for training. The dataset was divided into three subsets: 60% for training, 20% for validation, and 20% for testing. To augment the data and balance the classes with fewer images in the training and validation sets, the augmented images were generated for each original image in the cancer class. Expanding the dataset in this manner enhances the model’s robustness and generalizability while reducing the risk of overfitting [25]. The following data augmentation techniques were applied:

(1) Random horizontal flipping of images.
(2) Random vertical flipping of images.
(3) Random rotation of the images by angles of [0°, 90°, 270°].

### 3.4. Proposed Method

**Figure 1:**
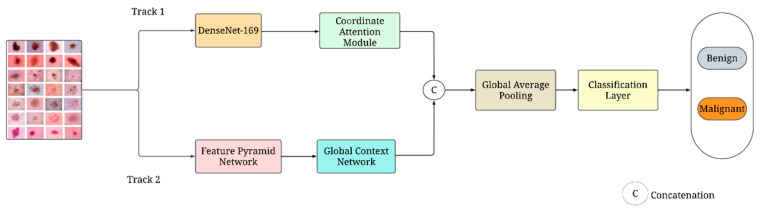
Overall architecture of the proposed network.

In this research, we introduce a novel dual-track DL network for skin lesion classification, comprising two distinct tracks: the first track employs a modified DenseNet-169 and the second track utilizes a customized CNN. Figure 1 illustrates the overall structure of the proposed network.

The DenseNet-169 architecture is enhanced by integrating a CoAM. This modification aims to improve the feature representation by emphasizing the relevant spatial features in the dermoscopic images. The output features from the modified DenseNet-169 are then forwarded to the CoAM to enhance the discriminative power of the extracted features. The custom CNN in the second track consists of two essential blocks: an FPN and a GCN. The FPN is designed to capture multi-scale features from the dermoscopic images, facilitating better representation of both local and global features. Subsequently, the GCN is incorporated to extract contextual information and enhance the feature discriminability. The following sections describe the proposed methods in detail.

#### 3.4.1. Track 1—Modified DenseNet

**Figure 2:**
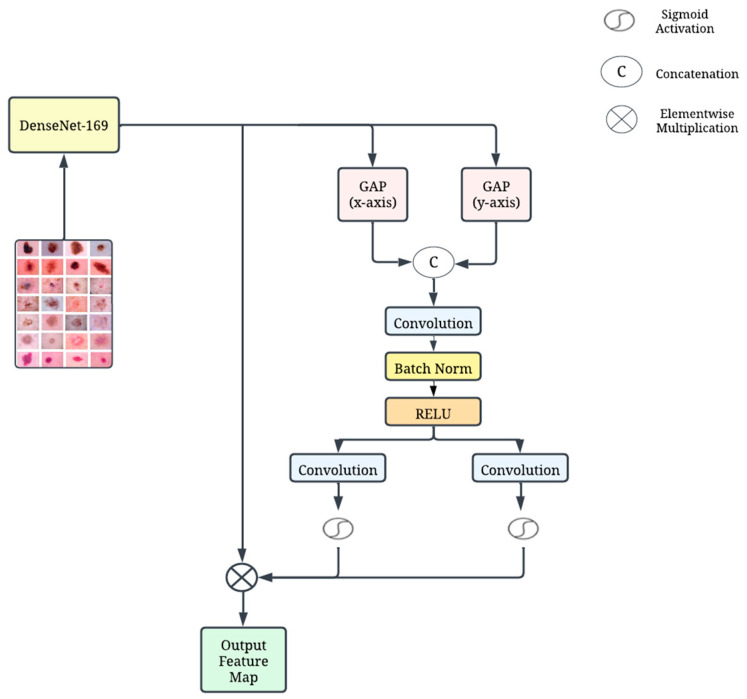
Schematic sketch of the track DenseNet with CoAM.

The architecture of modified DenseNet with CoAM is presented in Figure 2. DenseNet-169 is a CNN model that belongs to the family of DenseNets. One of its distinctive features is the dense connectivity pattern between layers, where every layer is connected to and receives input from all previous layers. This dense connectivity alleviates the vanishing-gradient problem and promotes reuse of features, enabling the network to be deeper and efficient in terms of parameter usage. Additionally, DenseNet-169 utilizes bottleneck layers to reduce the number of channels before the dense connectivity, which helps in reducing the computational cost and enhancing feature representation [26]. Between the dense blocks, transition layers with convolution and pooling operations are employed to downsample the feature maps and control the growth of feature maps. This approach helps to maintain a balance between the network’s representational power and computational efficiency. The features obtained from the DenseNet-169 architecture are then passed on to CoAM.

The CoAM is specifically designed to capture both granular local features and long-range dependencies within the input data [27]. This module is used to maintain important positional information, which is necessary for accurately capturing spatial features. In order to accomplish this, the average values are calculated for the channel of the feature map by taking the mean along both the horizontal (*x*-axis) and vertical (*y*-axis) dimensions using global average pooling. As shown in Figure 2, this is achieved by using two pooling kernels with different dimensions: (H, 1) and (1, W). The kernels encode each channel based on the horizontal as well as vertical coordinates, as shown in Equations (1) and (2).

(1) $$z_{c}^{h}\left(h\right)=\frac{1}{W}\sum_{0\leq i\leq W}x_{c}\left(h,i\right)$$

(2) $$z_{c}^{w}\left(w\right)=\frac{1}{H}\sum_{0\leq j\leq H}x_{c}\left(j,w\right)$$

By applying the two aforementioned formulas, information from both the height (*y*-axis) and width (*x*-axis) directions are effectively aggregated, resulting in the generation of direction-aware feature maps $z_{c}^{h}\left(h\right)$ and $z_{c}^{w}\left(w\right)$. These modifications enhance the capabilities of this module to accomplish two key objectives: capturing long-range dependencies within the data and simultaneously preserving critical positional information along the spatial dimensions. Consequently, the model’s ability to precisely locate features within the input is significantly enhanced.

Following concatenation, the features are passed through a convolutional layer for dimensionality reduction. This layer serves to decrease the number of channels within the feature representation, as expressed in Equation (3).

(3) $$f=\delta \left(F_{1}\left(\left[z^{h},z^{w}\right]\right)\right)$$

The notation [·, ·] denotes the concatenation operation performed along the spatial dimension. This operation combines the features resulting from Equations (4) and (5). Following concatenation, $f\;\in \;R^{C/r\times h}$, a non-linear activation function, is applied to the combined feature map (Z). This intermediate feature map, Z, encodes spatial information in both the horizontal and vertical directions. Subsequently, the feature map, Z, is split back into its two original groups along the spatial dimension. This essentially separates the information encoded for the horizontal and vertical directions. A convolution operation is applied within each group to alter the individual tensors while keeping the number of channels constant. This step refines the features within each directional group. Finally, a sigmoid operation is applied to the transformed features within each group. This operation serves to reweight the feature maps in both the x (horizontal) and y (vertical) positions. The mathematical formulations for these reweighting operations are presented in Equations (4) and (5).

(4) $$g^{h}=\sigma (F_{h}(f^{h}))$$

(5) $$g^{W}=\sigma (F_{W}(f^{W}))$$

Within this context, *σ* (sigma) represents the sigmoid function, a mathematical operation commonly employed for introducing non-linearity and transforming values into the range of 0 to 1. The notations *F_h_* and *F_w_* denote the output feature maps associated with each group, respectively, prior to the application of the transformation step. The feature map resulting from this process is formally defined in Equation (6).

(6) $$y_{c}\left(i,j\right)=x_{c}\left(i,j\right)\times g_{c}^{h}\left(i\right)\times g_{c}^{W}\left(j\right)$$

Here, *g^h^* and *g^w^* represent the output feature maps generated after the convolution operations performed within each group. These feature maps undergo a subsequent transformation process to calculate attention weights. The expanded versions of *g^h^* and *g^w^* are then utilized to selectively emphasize or suppress specific features within the input data, effectively guiding the model’s focus on informative regions.

The CoAM further enhances the features extracted by DenseNet-169, particularly focusing on extracting local features while preserving the positional information crucial for maintaining spatial characteristics. The CoAM encodes channels across the vertical and horizontal positions, allowing the module to effectively capture and maintain positional information across spatial dimensions. This combination of features and positional information enables the model to distinguish between different types of skin lesions based on their unique spatial and textural characteristics, thereby improving the performance of the classification process.

#### 3.4.2. Track 2—FPN and GCN

**Figure 3:**
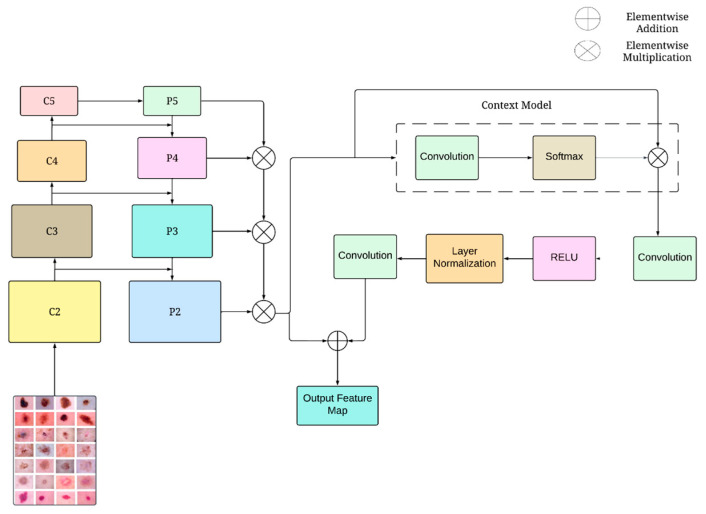
Schematic sketch of the blocks in the FPN and GCN.

In addition to the modified DenseNet-169 architecture in first track, the proposed network incorporates a second track featuring a customized CNN. This track is designed to capture and leverage the intricate features of skin lesions for improved classification accuracy. The CNN architecture in this track is tailored to integrate effective features extraction techniques, specifically an FPN and a GCN. The FPN enables the network to extract multiscale features, while the GCN focuses on aggregating global contextual information to enhance the feature representation. The overall architecture of track 2 is presented in Figure 3.

The FPN utilizes the hierarchical structure of a CNN to create a feature pyramid that includes high-level features consistently across different levels of semantics [28]. This method analyzes input images and generates feature maps of proportional sizes at various levels through a fully convolutional layer. The bottom-up pathway is the initial stage of the block, creating a hierarchy of features that includes feature matrices at different scales, with increments of 2. Each level generates output maps of identical dimensions. Following each stage’s residual block, the resulting feature maps are designated with specific labels: C2, C3, C4, and C5, corresponding to the outputs of the conv2, conv3, conv4, and conv5 layers, respectively. These feature maps are produced with strides of 4, 8, 16, and 32 pixels based on the input image. The dimensions of the feature maps produced by the FPN blocks C2, C3, C4, and C5 are 64 channels, 112 × 112 dimensions; 256 channels, 56 × 56 dimensions; 512 channels, 28 × 28 dimensions; and 1024 channels, 14 × 14 dimensions, respectively.

The top-down pathway enhances feature spatial resolution by upsampling feature maps from higher pyramid levels, despite their coarser spatial quality. It integrates elements from the bottom-up pathway through lateral connections, merging feature maps of the same spatial dimensions. The bottom-up feature map, containing basic features, offers more precise activation localization due to fewer subsampling stages. The feature map’s dimensions are doubled, starting from a lower resolution. The upsampled map is combined with the bottom-up map using a 1 × 1 convolutional layer to reduce channel size via element-wise addition. This iterative process continues until the most detailed map is formed. The final feature maps are labeled as {P2, P3, P4, P5}, corresponding to {C2, C3, C4, C5} in spatial dimensions, and are then consolidated and fed into the GCN.

GCNs are a specialized block integrated into the proposed architecture to enhance the model’s ability to extract global contextual information within input images [29]. Unlike standard CNN filters, GCNs utilize larger receptive fields to incorporate information from a wider area, enabling them to capture complex relationships between distant regions. This is particularly valuable for tasks like analyzing medical images. In the context of skin cancer classification using dermoscopic images, GCNs play a crucial role. By extracting long-range dependencies, they provide subsequent network layers with a more comprehensive understanding of the entire input image. This ability to capture the spatial distribution of abnormalities and the relationships between different skin regions is essential for accurate diagnosis. Additionally, the global context-based features extracted by GCNs can be interpreted to identify spatial patterns within the image, facilitating a deeper exploration of the disease and potentially revealing insights into its progression. By leveraging GCNs, the proposed model explores global spatial information, enables the inclusion of broader contextual information, and improves the performance in skin cancer detection.

Within the GC block, the non-local network leverages a weighted summation of features across the entire spatial extent of the feature matrix to compute the output for each individual position. This mechanism enables the network to capture inter-regional or inter-object relationships within the image, refining its capacity to generate context-sensitive predictions. The GCN module commences by applying a 1 × 1 convolution followed by a softmax function. This operation yields attention weights and contextual features. Subsequently, the attention weights are utilized to process the initial features, then combines them with information about the entire image. To generate a channel-wise attention map, the GCN module proceeds with a spatial compression of the feature map using a 1 × 1 convolution. This is followed by layer normalization, ReLU activation, and a concluding 1 × 1 convolution. Finally, the feature vector of the GCN module is concatenated with the original feature map to incorporate the global context information. Modeling the global context is defined by Equation (7):

(7) $$P_{i}=F\left(I_{i},\delta \left(\sum_{j=1}^{n}\alpha_{j}I_{j}\right)\right)$$

where, $\alpha_{j}=\frac{e^{W_{s}I_{j}}}{\sum_{z}^{R_{p}}e^{W_{s}I_{m}}}$ denotes the weight associated with global attention pooling. ‘*I*’ represents the feature map extracted from the FPN. ‘*W_s_*’ signifies the transformation matrix, and ‘*n*’ refers to the total number of positions within the feature matrix.

The $\sum_{j=1}^{n}\alpha_{j}I_{j}$ is the context module within network architecture. This module accomplishes feature fusion by applying a weighted averaging operation across all spatial locations in the feature matrix. The weights for this operation are denoted by *α_j_*. This process culminates in the extraction of global contextual features. The notation δ(.) is a transformation function specifically designed to extract dependencies across feature channels. F(.,.) is a function employed to aggregate the aforementioned global contextual features with the features associated with all the positions within the feature map.

The proposed architecture utilizes a more detailed feature representation of the input data. This is achieved by passing the improved features extracted by the FPN to the subsequent GCN module. The GCN module specifically focuses on extracting crucial global context features. The FPN is adept at capturing multi-scale features within the input data. Subsequently, the GCN module refines these features by incorporating the aforementioned global contextual information. This collaborative approach empowers the model to effectively utilize both local and global features, ultimately leading to a more discriminative and robust feature representation. The feature maps, enhanced with global context information, are passed to the following layers within the network architecture for further processing and classification tasks.

#### 3.4.3. Classification

The feature maps obtained from both the tracks are combined to leverage the complementary strengths of both networks. The fused features are then fed into a classification layer, which performs the final classification of the skin lesions into different categories. The fusion of features from both tracks allows the model to integrate both local features extracted by DenseNet-169 and global multiscale features with contextual information extracted by FPN and GCN. This feature fusion enables the model to make more informed and accurate decisions by considering both local details and global context, leading to improved classification results. This process facilitates a deeper understanding of the inter-regional and inter-object relationships present within the input data. This refined feature representation contributes to improved semantic comprehension and leads to more effective decision making by the model. Global average pooling is used to decrease the spatial dimensionality of the refined features retrieved from the feature maps. This technique effectively reduces the overall number of parameters within the network architecture, consequently leading to a significant decrease in the model’s computational overhead. The pooled features are subsequently forwarded through a flattening layer and a dropout layer. This serves to regularize the model and prevent overfitting. The features then proceed to a fully connected hidden layer and a ReLU activation function. An additional dropout layer is implemented for further regularization. The final features are then inputted to the classification layer responsible for assigning class labels to the input data. To optimize and update the model weights during the training phase, focal loss is employed as the loss function [30]. It helps in addressing the challenge of class imbalance by modifying the standard cross-entropy loss function. This approach not only fosters enhanced classification accuracy but also guides the model in fine-tuning its weights and biases, facilitating more precise classification across a diverse range of classes.

## 4. Experimental Results and Discussion

This section provides the experimental findings to assess the performance and effectiveness of the proposed dual-track architecture for skin cancer classification. It includes ablation studies to assess the contributions of each block, as well as comparisons with state-of-the-art networks and existing works.

### 4.1. Ablation Studies

To systematically evaluate the contribution of each block and track in the proposed model, a series of ablation studies were conducted. These studies aimed to assess the effectiveness of the modified Densenet-169 architecture, the integration of the CoAM, and the performance of the FPN and GCN in the customized CNN architecture.

#### 4.1.1. Effectiveness of the Densenet-169 Network

To evaluate the effectiveness of the modified Densenet-169 architecture in first track, the experiments were conducted to compare its performance with a baseline model using a standard Densenet-169 architecture. Following feature extraction, the output feature maps were subjected to global average pooling for dimensionality reduction along the spatial axes. Then the feature maps were fed into a flattening layer and classification layers.

**Figure 4:**
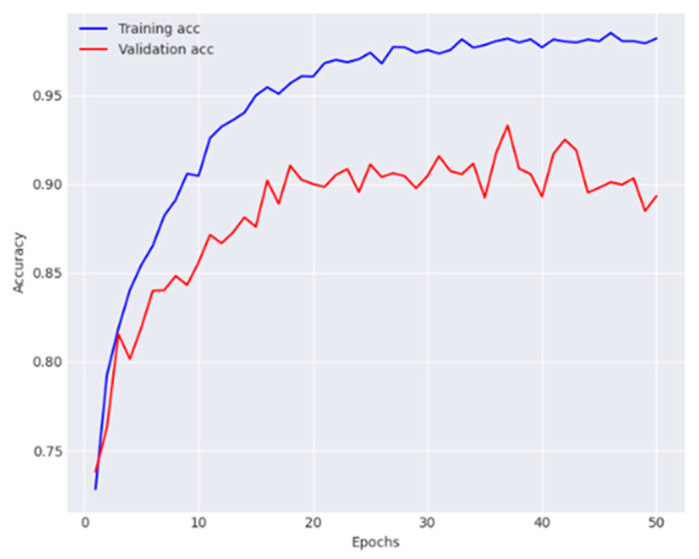
Analysis of training phase of the DenseNet-169 in terms of accuracy.

**Figure 5:**
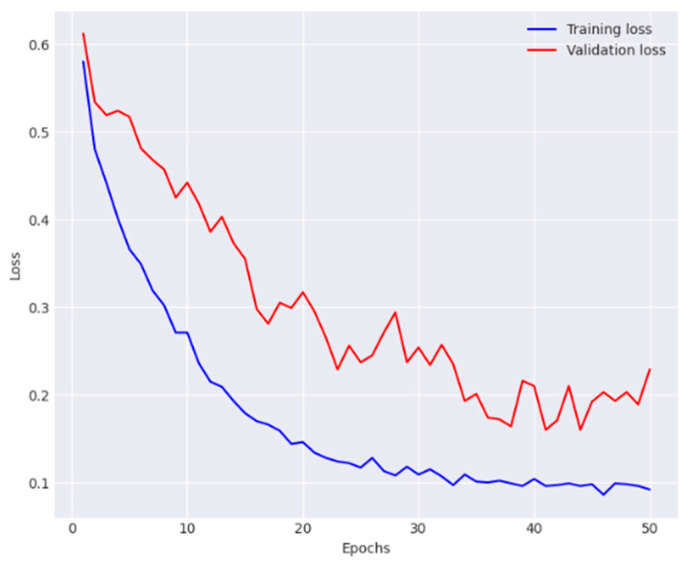
Analysis of training phase of the DenseNet-169 in terms of loss.

Figure 4 and Figure 5 illustrate the model training and validation process. Furthermore, the network achieved an accuracy of 85.7%, average precision of 85.9%, an average recall of 86.1%, and an average F1-score of 86%. The performance metrics obtained for each class are presented in Table 2.

#### 4.1.2. Effectiveness of the Densenet-169 with CoAM

**Figure 6:**
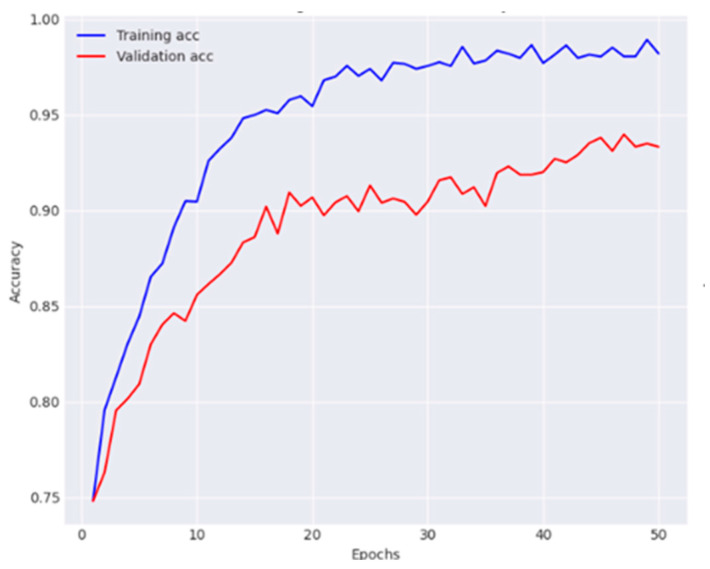
Analysis of training phase of the DenseNet and CoAM in terms of accuracy.

**Figure 7:**
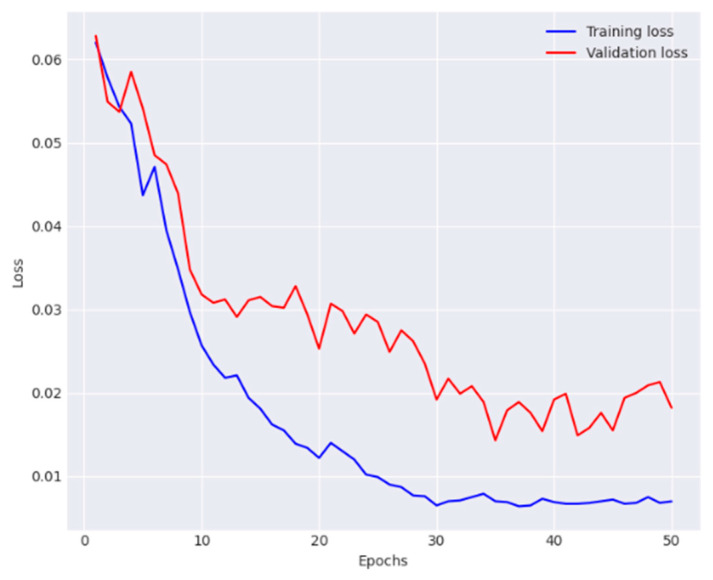
Analysis of training phase of the DenseNet and CoAM in terms of loss.

We further investigated the effectiveness of combining the CoAM with the modified Densenet-169 architecture. The experiments showed that the addition of CoAM improved the feature extraction capabilities of the Densenet-169, leading to enhanced classification accuracy. The CoAM was found to effectively extract local features and maintain positional information, contributing to the improved performance of the model. Figure 6 and Figure 7 present the training and validation observations made by the network.

The DenseNet-169 model with the CoAM block achieved an accuracy of 90.5% on the testing set. Furthermore, the model attained a precision of 91.4%, a recall of 90.6%, and an F1-score of 91%. The evaluation metrics for each class are provided in Table 3.

#### 4.1.3. Effectiveness of the FPN and GCN Blocks

To assess the effectiveness of the customized CNN architecture in Track 2, we conducted experiments to evaluate the performance of the FPN and GCN individually and in combination. The results indicated that both the FPN and GCN contributed to the improved feature representation and classification accuracy. The combination of FPN and GCN led to a synergistic effect, further enhancing the model’s performance. Integrating the GCN module with the feature extraction capabilities of the FPN block enhances the network’s capacity to process information. This is achieved by focusing on the most informative regions within the input data while simultaneously suppressing features of lesser relevance.

**Figure 8:**
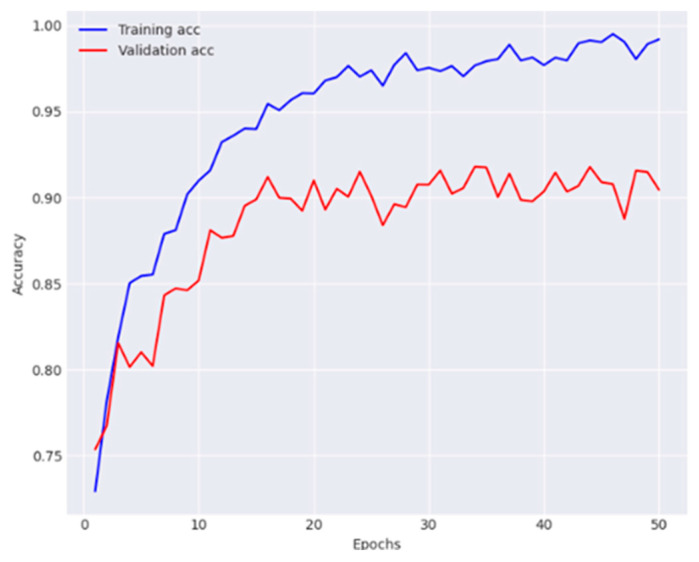
Analysis of training phase of the FPN and GCN in terms of accuracy.

**Figure 9:**
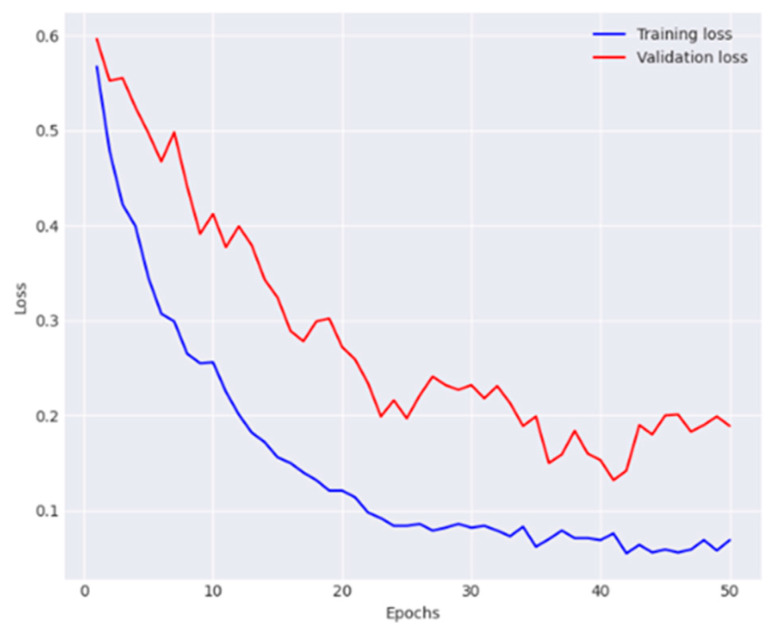
Analysis of training phase of the FPN and GCN in terms of loss.

The performance metrics obtained for each class are provided in Table 4. The model reported 91.0% average accuracy, 89.8% average recall, 92.5% average precision, and 91.1% average F1-score. Training and validation observations are presented in Figure 8 and Figure 9.

#### 4.1.4. Effectiveness of the Proposed Network

**Figure 10:**
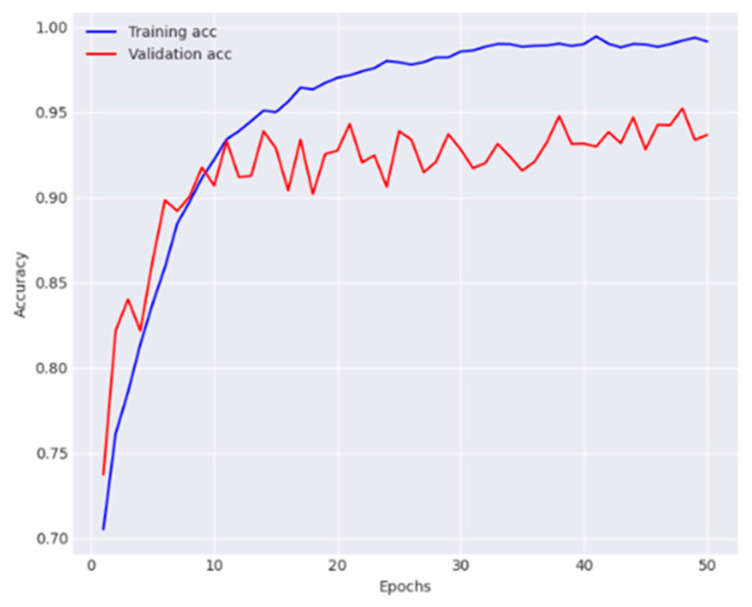
Analysis of the proposed model in terms of accuracy.

**Figure 11:**
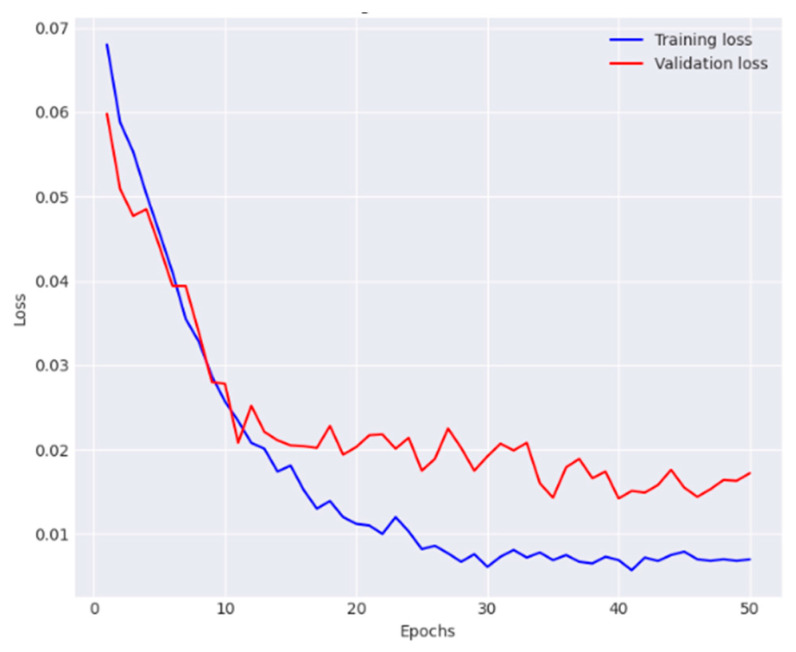
Analysis of the proposed model in terms of loss.

Finally, the overall effectiveness of the proposed dual-track deep learning model was evaluated, which integrates the modified Densenet-169 from first track and the customized CNN with FPN and GCN from track 2. The experimental findings show that the proposed technique outperformed the individual tracks and baseline models, confirming the effectiveness of the dual-track architecture for skin lesion classification. The observations of training and validation are presented in Figure 10 and Figure 11.

The proposed model reported 93.2% average accuracy, 91.4% average recall, 95.3% average precision, and 93.3% F1-score. The performance metrics obtained for each class are provided in Table 5. This table offers granular details regarding the model’s classification accuracy for each individual category within the dataset. The results of the conducted ablation studies are summarized in Table 6. It elucidates the contribution of various components within the proposed architecture to its overall effectiveness.

### 4.2. Performance Comparison with State-of-the-Art Networks

The performance of the proposed network was compared with the state-of-the-art networks for skin lesion classification using HAM10000 dataset. The results show that the proposed method outperformed other networks in terms of classification accuracy, demonstrating its effectiveness for skin cancer classification. Figure 9 offers a concise visualization of the evaluation results obtained on the test set. Among the pre-trained models evaluated, DenseNet-169 emerged as the most accurate, achieving a score of 85.7%. Notably, DenseNet-169 with a score of 85.3% closely followed this performance. Conversely, ResNet-18 exhibited a lower accuracy of 79.6%. Furthermore, when the proposed model was implemented using DenseNet-169 as its foundation, a significant accuracy improvement of 7.5% was observed. Table 7 provides a detailed breakdown of these results.

### 4.3. Performance Comparison with Existing Works

Additionally, the performance of the proposed architecture was compared with the existing works that utilize similar datasets and evaluation protocols. The comparative analysis revealed that the proposed method achieved significant improvements in classification accuracy, robustness, and generalization capabilities compared to existing works, further validating the effectiveness of the proposed dual-track DL model for skin lesion classification. Table 8 presents a comparative analysis of the proposed approach with established DL techniques employed for binary classification tasks. The referenced studies encompass a diverse range of methodologies, including transfer learning and CNN, which highlights the ongoing exploration of various DL architectures within this domain. Notably, the proposed model achieves the highest accuracy metric as reported in the table. This accomplishment underscores the significant potential of custom-designed DL models to demonstrably improve the classification of skin cancer, ultimately contributing to enhanced medical diagnostics and more effective patient care.

### 4.4. Limitations and Future Works

1. Additional validation using diverse datasets is required to substantiate the model’s ability to generalize effectively. External validation on independent datasets is imperative to validate its robustness across diverse clinical settings.
2. In order to enhance the applicability and efficacy of skin cancer detection in practical settings, forthcoming studies will integrate supplementary features, including patient metadata, as well as region-specific and gender-specific analyses.
3. This work will be expanded by employing a more extensive dataset, thereby increasing the diversity of the sample population.

## 5. Conclusions

In this study, a novel dual-track deep learning model for skin lesion classification is proposed, integrating a modified Densenet-169 architecture with a customized CNN featuring an FPN and GCN. The first track of the proposed model integrates Densenet-169 architecture with CoAM to improve feature extraction capabilities. The second track consists of two blocks: the FPN captures multiscale features, and the GCN aggregates global contextual information. The local features extracted from first track and global features extracted from second track are fused and passed to a classification layer for final classification. By using this approach, the model benefits from the pre-trained knowledge while incorporating adaptations specific to the classification of skin lesion. The experimental findings show the efficacy of the proposed dual-track DL model for skin cancer classification. An evaluation using the HAM10000 dataset yielded an accuracy of 93.2% for the proposed network. These findings hold considerable significance for the field of dermoscopic image-based diagnostics. The proposed model also offers promising new avenues for the identification and management of cancerous skin lesions.

## Figures and Tables

**Table 1:** Tuning of hyperparameters for model training.

Parameter	Search Space	Optimal Value
Weight Decay	[0, 1 × 10^−3^, 1 × 10^−4^, 1 × 10^−5^]	1 × 10^−3^
Learning rate	[1 × 10^−3^, 1 × 10^−4^, 1 × 10^−5^]	1 × 10^−4^
Batch Size	32 or 64	32
Dropout	[0–0.33]	0.15
Epochs	[30–100]	50
Optimizer	[SDG, BGD, ADAM]	ADAM

**Table 2:** Class-wise evaluation metrics for the DenseNet-169 architecture.

Class	Accuracy (in %)	Precision (in %)	Recall (in %)	F1-Score (in %)
Benign	86.1	85.9	86.1	86.6
Malignant	85.2	85.4	85.2	85.3

**Table 3:** Performance evaluation for the DenseNet-169 with CoAM.

Class	Accuracy (in %)	Precision (in %)	Recall (in %)	F1-Score (in %)
Benign	90.6	91.4	90.6	91.0
Malignant	90.5	89.6	90.5	90.0

**Table 4:** Class-wise evaluation metrics obtained for track 2 blocks.

Class	Accuracy (in %)	Precision (in %)	Recall (in %)	F1-Score (in %)
Benign	92.2	92.5	92.2	91.1
Malignant	89.8	89.5	89.8	90.8

**Table 5:** Performance evaluation metrics obtained for proposed method.

Class	Accuracy (in %)	Precision (in %)	Recall (in %)	F1-Score (in %)
Benign	95.1	95.3	95.1	93.3
Malignant	91.4	91.4	91.4	93.1

**Table 6:** Summary of the ablation study results.

Experiments	Accuracy in (%)	Precision in (%)	Recall in (%)	F1-Score in (%)
DenseNet-169	85.7	85.9	86.1	86.0
DenseNet-169 with CoAM	90.5	91.4	90.6	91.0
FPN with GCN	91.0	92.5	89.8	91.1
Proposed network	93.2	95.3	91.4	93.3

**Table 7:** Performance evaluation of the proposed network with state-of-the-art networks.

S. No.	Model Trained	Accuracy in %
1	ResNet-18	79.6
2	ResNet-50	82.1
3	VGGNet-16	82.2
4	ResNet-34	83.4
5	ResNet-152	83.7
6	DenseNet-121	83.8
7	AlexNet	84.6
8	DenseNet-201	85.3
9	DenseNet-169	85.7
10	Proposed network	93.2

**Table 8:** Evaluating the performance of the proposed architecture against existing works.

S. No	Source	Method	Accuracy
1	Bechelli et al. [31]	Custom CNN	82
2	Huang et al. [17]	Custom CNN	85.8
3	Mehr et al. [23]	Custom CNN	89.3
4	Ali et al. [32]	Transfer Learning	91.3
5	Salamaa et al. [21]	Transfer Learning	92
6	Hekler et al. [33]	Fusion method	82.95
7	Mrindha et al. [34]	Custom CNN	92.1
8	Ameri A. [35]	Transfer Learning	84
9	Proposed Method	Custom CNN	93.2

## Data Availability

The HAM10000 dataset is available at: https://dataverse.harvard.edu/dataset.xhtml?persistentId=doi:10.7910/DVN/DBW86T (accessed on 27 January 2024).
